# Multi role ChatGPT framework for transforming medical data analysis

**DOI:** 10.1038/s41598-024-64585-5

**Published:** 2024-06-17

**Authors:** Haoran Chen, Shengxiao Zhang, Lizhong Zhang, Jie Geng, Jinqi Lu, Chuandong Hou, Peifeng He, Xuechun Lu

**Affiliations:** 1 School of Management, Shanxi Medical University, Taiyuan, 030000 China; 2 Department of Nephrology, First Medical Center of Chinese PLA General Hospital, Nephrology Institute of the Chinese People’s Liberation Army, National Key Laboratory of Kidney Diseases, National Clinical Research Center for Kidney Diseases, Beijing Key Laboratory of Kidney Disease Research, Beijing, 100853 China; 3 Department of Rheumatology and Immunology, The Second Hospital of Shanxi Medical University, Taiyuan, China; 4 Key Laboratory of Coal Environmental Pathogenicity and Prevention at Shanxi Medical University, Ministry of Education, Taiyuan, Shanxi China; 5 Basic Medicine College, Shanxi Medical University, Taiyuan, 030000 China; 6 Department of Computer Science, Boston University, 665 Commonwealth Avenue, Boston, MA 02215 USA; 7 Department of Hematology, The Second Medical Center of Chinese PLA General Hospital, National Clinical Research Center for Geriatric Disease, Beijing, 100853 China; 8 Medical School of Chinese PLA, Beijing, 100853 China; 9 Shanxi Key Laboratory of Big Data for Clinical Decision, Shanxi Medical University, Taiyuan, 030000 China; 10 SXMU-Tsinghua Collaborative Innovation Center for Frontier Medicine, Shanxi Medical University, Taiyuan, China

**Keywords:** ChatGPT, Automation, Bioinformatics, Transcriptome, Data integration, Data mining, Databases, Target identification

## Abstract

The application of ChatGPTin the medical field has sparked debate regarding its accuracy. To address this issue, we present a Multi-Role ChatGPT Framework (MRCF), designed to improve ChatGPT's performance in medical data analysis by optimizing prompt words, integrating real-world data, and implementing quality control protocols. Compared to the singular ChatGPT model, MRCF significantly outperforms traditional manual analysis in interpreting medical data, exhibiting fewer random errors, higher accuracy, and better identification of incorrect information. Notably, MRCF is over 600 times more time-efficient than conventional manual annotation methods and costs only one-tenth as much. Leveraging MRCF, we have established two user-friendly databases for efficient and straightforward drug repositioning analysis. This research not only enhances the accuracy and efficiency of ChatGPT in medical data science applications but also offers valuable insights for data analysis models across various professional domains.

## Introduction

In its second-year post-release, ChatGPT, a prominent Large Language Model (LLM), has significantly influenced various fields. However, the effectiveness and applicability of ChatGPT in certain areas are still skeptical, especially for mission-critical domains like health sciences^1,2^. Concerns have emerged regarding the adequacy of prompts to extract accurate information, strategies to ensure result consistency, standards for quality control (QC) of responses, and methods to mitigate errors arising from ChatGPT's inherent randomness. In our previous data mining endeavors, we encountered challenges stemming from heavy reliance on human and material resources, resulting in high costs and reduced efficiency. The expansive nature of research projects and the diverse yet sometimes limited expertise of team members further compounded these challenges.

To overcome these limitations, we developed a Multi-Role ChatGPT Framework (MRCF), which enables ChatGPT to assume diverse roles in scientific research, significantly enhancing speed, reducing costs, and improving accuracy and consistency in data analysis. Our primary application of the MRCF has been in the automated mining of the GEO database, a task for which the framework has been specifically optimized to handle large-scale data categorization and analysis efficiently. We have also established two databases: the GEO Auto-Classification Database (https://chat.bio-db.com/GACdb) and the Drug Repurposing Database (https://chat.bio-db.com/DrugOntoDB/). The effective performance of these databases underscores the substantial value of the MRCF framework.

## Results

### Overview

**Figure 1:**
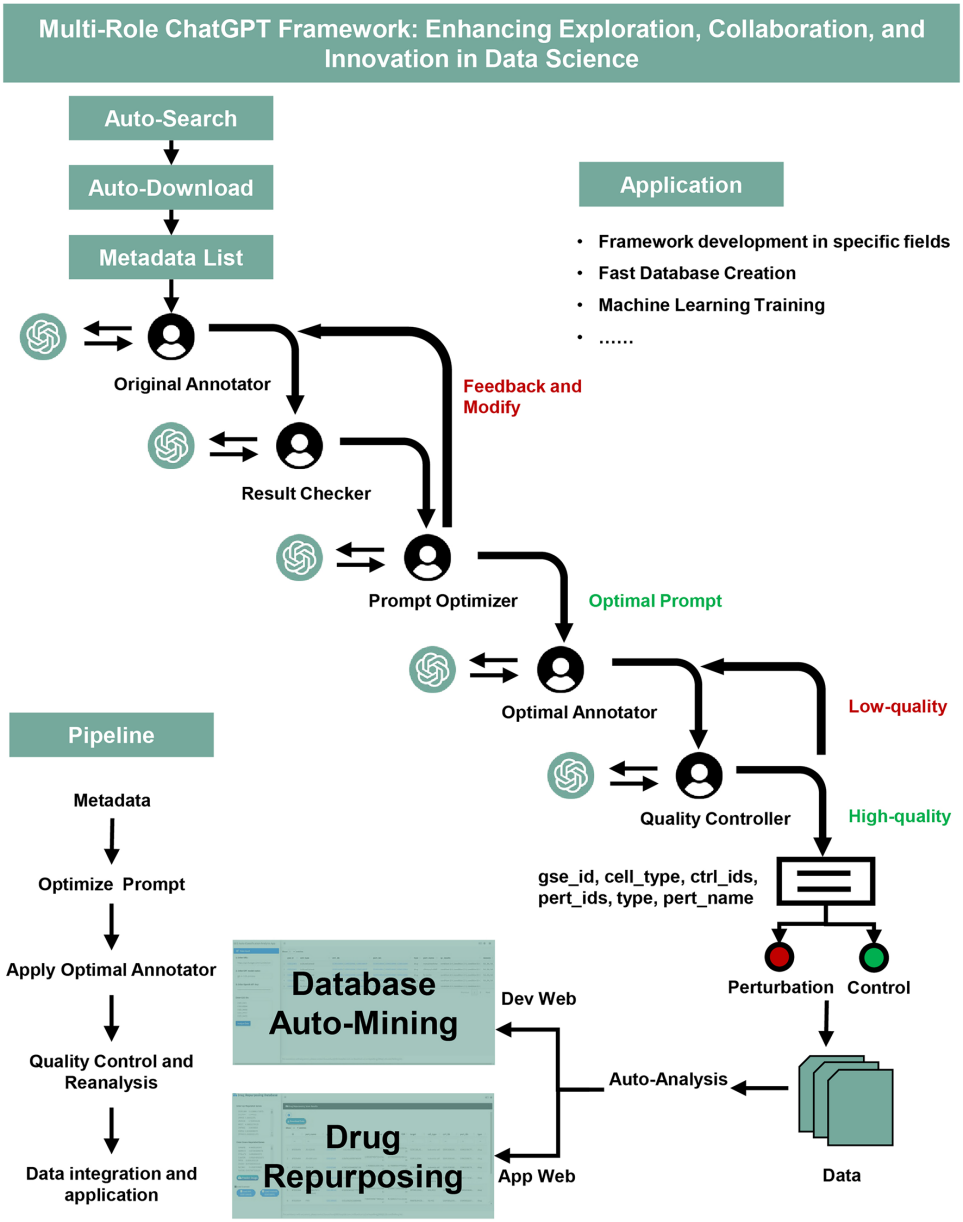
The Workflow of the Multi-Role ChatGPT Framework integrates the Waterfall Method to delineate data flow. This flexible framework suits a wide range of data science analytics applications using ChatGPT to perform each task.

To enhance the accuracy of data annotation, we have developed the Model Role Configuration Framework (MRCF) based on Standardized Operating Procedures (SOP), as depicted in Fig. 1. This framework assigns distinct roles to each participant in the ChatGPT setup, optimizing the workflow to improve efficiency and accuracy. Initially, the *Original Annotator* pre-annotates the data. These annotations are then scrutinized by the *Result Checker*, who compares them against a gold standard to spot discrepancies. Upon finding discrepancies, the *Prompt Optimizer* suggests modifications, which are resubmitted to the *Original Annotator*. This process is repeated until the *Optimal Annotator* is identified, which is then utilized to apply this refined annotator to other datasets. The outcomes are subsequently reviewed by the *Quality Controller*.

The *Quality Controller* ensures that the data annotation results conform to three specific criteria that uphold the project's stringent standards. Firstly, it is imperative that there is a strict alignment with GSM Characteristics; this involves double-checking the GSM details for both control_ids (control sample GSM ID) and pert_ids (perturbation sample GSM ID) to confirm that they match their respective GSM characteristics. Here, we ensure that all variables, except sex and age, which are expressly ignored, remain constant between pert_ids and ctrl_ids. Secondly, uniformity is maintained by consolidating all samples of the same type into a single group. Lastly, it is crucial to verify that perturbation details such as the type of perturbation (drug, disease, or gene), cell type, and pert_name (perturbation name) are correctly assigned and clearly distinguished between pert_ids and ctrl_ids. This rigorous process facilitates the automation of data analysis and the construction of a professional database that meets established quality standards.

To be specific, we utilized 338 manually annotated drug datasets from the CREEDS (CRowd Extracted Expression of Differential Signatures) database as the gold standard for evaluation^3^. Through iterative training, we developed the *Optimal Annotator*, which was then employed to annotate 8730 transcriptome sequencing datasets related to 782 drugs in the GEO (Gene Expression Omnibus) database^4^ (see Supplementary Table S1). In the preliminary assessment of the framework, we selected 964 datasets for annotation, QC, and performance evaluation. Finally, we created two user-friendly databases: the GEO Auto-Classification Database for automated annotation of GEO datasets and the Drug Repurposing Database aimed at predicting drugs with similar or opposing gene expression patterns.

### Optimization and evaluation of prompts

**Figure 2:**
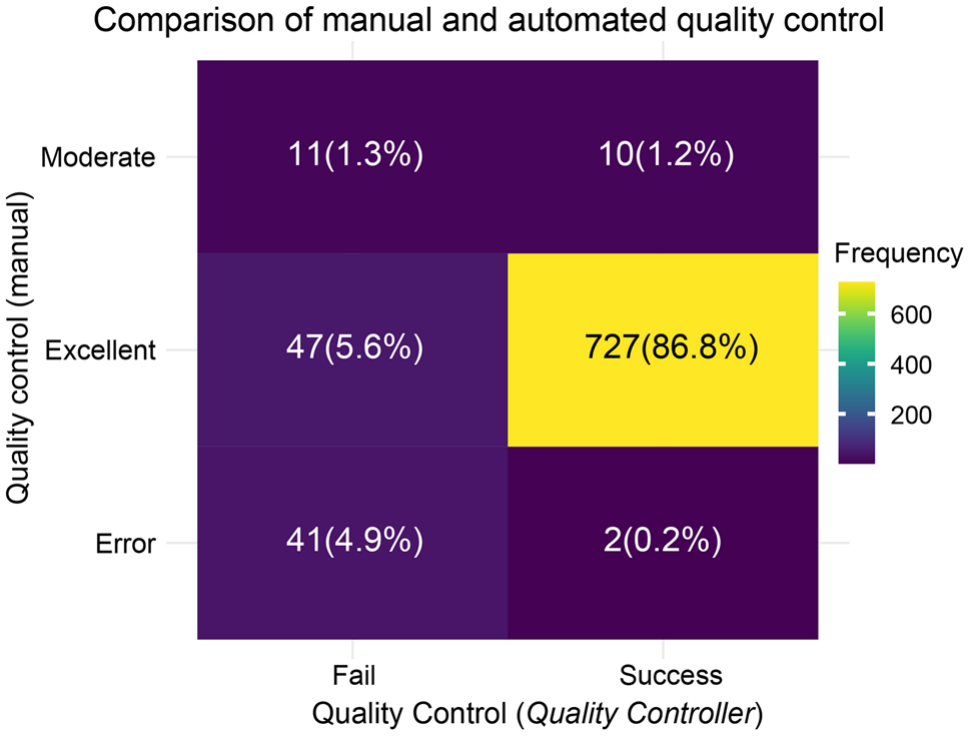
Comparison of manual and *Quality Controller* QC results.

We analyzed 317 CREEDS datasets using the *Optimal Annotator*, resulting in 838 annotations. The QC outcomes were categorized into three levels: 'Excellent' (where ChatGPT annotations surpassed manual annotations, as shown in Supplementary Table S1 for GSE10192), 'Moderate' (annotations were essentially consistent or contained ambiguous information), and 'Error' (annotations with errors). Concurrently, we employed the *Quality Controller* for automatic QC, categorized as 'Success' (meeting three criteria) or 'Fail' (not meeting the criteria). 91.7% (86.8% + 4.9%) of the annotations were consistent in both automatic and manual QCs. For the remaining 8.3% (5.6% + 1.3% + 1.2% + 0.2%) annotations with errors, we conducted a detailed analysis and identified the causes to be multiple disturbances, sample age differences, or inconsistencies in disturbance names among others(Fig. 2). These causes of error have been documented in Supplementary Table S1. Overall, these findings underscore the robustness of the *Optimal Annotator* and the significance of implementing a *Quality Controller*.

### Practical implementation and evaluation of the multi-role ChatGPT framework

Throughout the entire process, we focused on three key evaluation metrics: Quality Control Consistency (QCC), Annotation Pass Rate (APR), and Dataset Pass Rate (DPR). QCC refers to the proportion of consistency between two QC outcomes; APR is the proportion of annotations that pass the QC criteria; DPR, on the other hand, assesses the proportion of datasets that pass QC standards out of the total datasets reviewed. Additionally, given the diversity of experimental designs within a single dataset, we have established a criterion where a dataset is considered to have passed if it contains at least one annotation that conforms to our defined quality standards.

**Figure 3:**
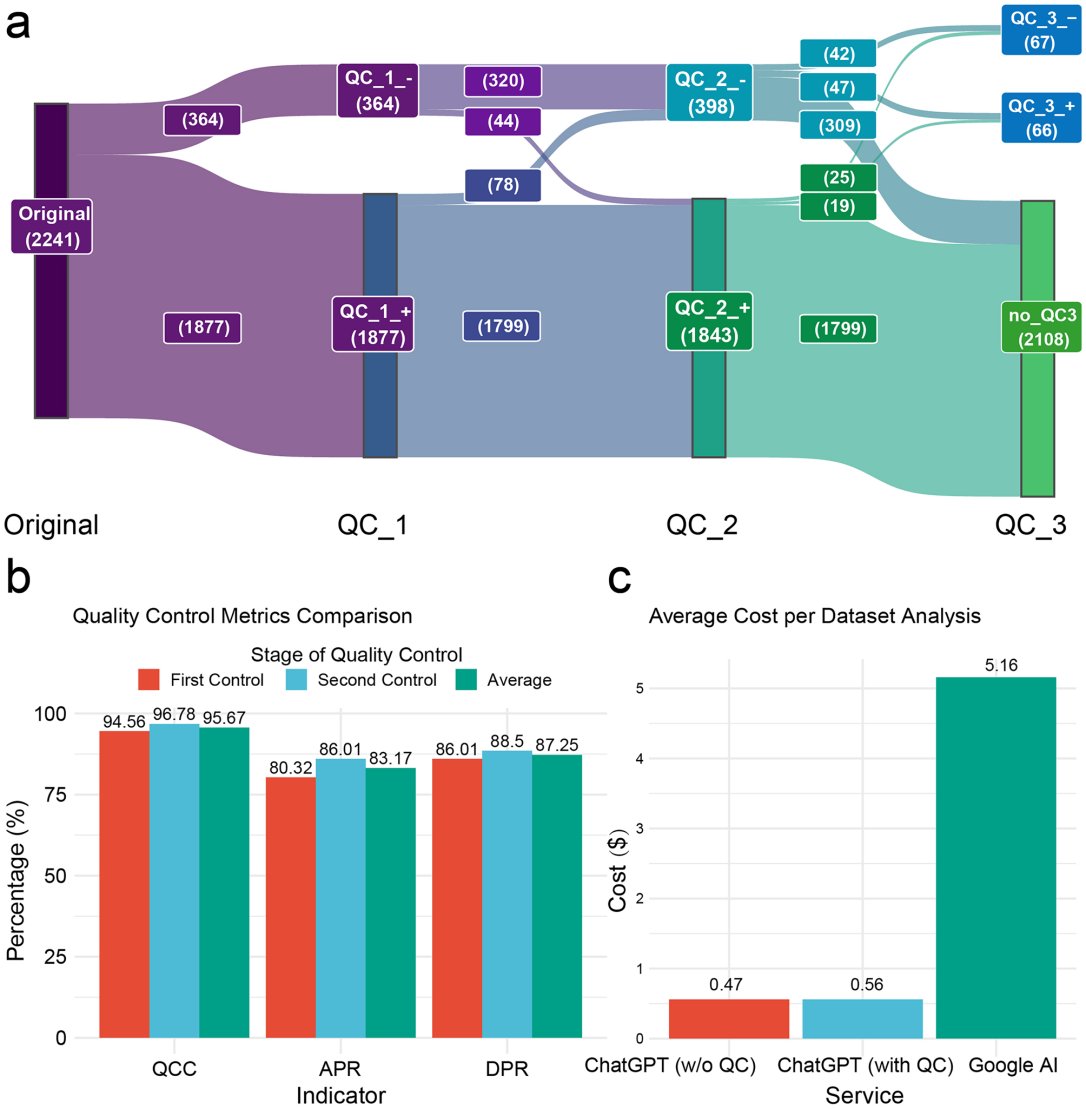
Performance Display of the Multi-Role ChatGPT Framework. (**a**) Distribution of 2241 annotations results in the workflow, where *QC_1_-*represents annotations that failed in the 1st QC. (**b**) Performance of QCC, APR, and DPR in the framework. (**c**) Cost comparison per dataset between MRCF and Google AI Platform's data annotation service, based on the calculation for each dataset containing 2000 words (including abstracts, titles, descriptions, and sample characteristics).

Analysis of errors in the CREEDS data revealed issues not only with the prompts but also with the inherent complexity of the datasets themselves. Therefore, we incorporated QC and negative feedback mechanisms into our study to correct avoidable errors. Analysis of 964 datasets from the GEO database indicated that the first round of annotation yielded 2,241 results, with the *Optimal Annotator* and *Quality Controller* demonstrating high consistency and acceptance rates in three rounds of QC (Fig. 3a,b, Supplementary Table S3). Additionally, compared to traditional methods of similar data annotation, MRCF resulted in a 90% cost reduction (see Fig. 3c). The average analysis time of the MRCF for each dataset was only 3 s (approximately 2000 words), whereas experts typically require half an hour to complete the same task.

For the 133 annotations where the two QCs were inconsistent, a second annotation was conducted, with the APR dropping to 47.76% (Supplementary Table S4). This reveals the importance of selecting appropriate datasets and adhering to strict QC standards. Datasets that failed to pass QC even after multiple annotations also underscore their inherent limitations.

In summary, the MRCF exhibits high accuracy, strong QC consistency, cost-effectiveness, and efficiency, making it suitable for large-scale data mining in complex professional fields.

### Automated differential expression analysis processing

The GEO database encompasses datasets of expression profiles for all microarrays and some RNA-seq. To supplement the RNA-seq datasets, we incorporated the GREIN database (http://www.ilincs.org/apps/grein), ultimately obtaining 624 datasets with expression matrices, yielding 1411 annotated results. To identify differentially expressed perturbation genes, we applied automated and standardized analysis procedures to both microarray and RNA-seq, and these results contributed to the construction of the Drug Repurposing Database.

### Construction of user-friendly databases

To facilitate use by researchers, we developed the user-friendly GEO Auto-Classification Database (https://chat.bio-db.com/GACdb). The complete MRCF analysis can be conducted without programming skills, and results are stored using the RSQLite package for efficient, repeat access. This enables small-scale research teams to rapidly construct large databases in specific medical fields.

In contrast to the CREEDS database, which relied on contributions from over 70 volunteers across 25 countries to manually annotate about 4185 perturbations across various categories, GACdb automates the annotation of all 170,000 transcriptome datasets in the GEO database. This not only vastly expands the scope and volume of data processed but also significantly enhances the efficiency and accuracy of annotations. By automating quality control and reducing reliance on manual input, GACdb delivers annotations at a fraction of the time and cost associated with traditional methods, providing a substantial advancement in the field of bioinformatics.

We also created the Drug Repurposing Database (https://chat.bio-db.com/DrugRepurposing/) using quality-controlled perturbation datasets. This database is designed to identify perturbations with similar or opposing gene expression patterns and to screen potential targets. In the aspect of disease perturbation prediction, we found significant negative correlations between acute myeloid leukemia (AML) and drugs like JS-K, γ-interferon, dubermatinib (TP-0903), and gilteritinib (Supplementary Table S5). Studies have shown that JS-K induces cancer cell apoptosis through the release of nitric oxide (NO), γ-interferon has immunomodulatory and anti-tumor cell proliferation effects, TP-0903 selectively inhibits Axl receptor tyrosine kinase, and gilteritinib has specific therapeutic effects on various FLT3 mutations^5–8^. Conversely, drugs like infliximab and flurbiprofen showed significant positive correlations with the gene expression patterns of AML. Studies indicate that infliximab, a TNFα blocker, can increase the risk of leukemia and lymphoma, possibly by reducing T-cell recognition of cancer cells and increasing the risk of tumor cell proliferation and metastasis^9^. Another study confirmed that flurbiprofen might increase the relative risk of AML in women^10^. In terms of drug perturbation prediction, our analysis showed significant positive correlations in the gene expression patterns of imatinib with dasatinib, erlotinib, and an independent dataset of imatinib, all of which are tyrosine kinase inhibitors^11^. In contrast, significant negative correlations were found with infliximab, thalidomide, and hydrocortisone. Studies have shown that the concurrent use of imatinib with these drugs reduces neutrophil levels and increases infection risk^12^. In medical research, there is often contradictory evidence, particularly regarding drug treatment effects and side effects. To address this challenge, our Drug Repurposing Database can accurately analyze and identify the suitability of specific patients for drugs and screen alternative medications, providing precise medical decision suggestions.

## Discussion

### ChatGPT in biomedicine: challenges and innovations

In the field of biomedicine, although ChatGPT can pass certain exams, it often generates incorrect responses that fail to meet the requirements, and this inconsistency limits its application in clinical research^13,14^. To address this, we innovatively developed the MRCF, standardizing the input and output of each role through SOP and evaluating framework performance. Our GEO Auto-Classification Database, developed based on this framework, outperforms the conventional ChatGPT (gpt4-preview-1106 version) workflow and manual annotations in terms of consistency, accuracy, speed, and cost. Meanwhile, the Drug Repurposing Database aids in clinical decision making, drug screening, and target prediction. Overall, this framework possesses the capability to facilitate research across a wide range of problems and also holds significant potential for application in various fields.

Our research aligns with Truhn’s view: “Large language models should be used as scientific reasoning engines, not knowledge databases”^15^. Our pipeline uses metadata from the actual GEO database, not the built-in knowledge base of ChatGPT. By analyzing standardized metadata, our framework generates reasoning results based on real data rather than “hallucinations.” Such a process can establish standardized databases for real-world data mining and clinical applications.

Ali et al. used ChatGPT to write patient clinical letters, as shown in the supplementary materials S1 of their study^16^. They used their own defined prompts for queries but overlooked the accuracy or completeness of prompts. We iteratively refined the *Original Annotator* with training sets and negative feedback, resulting in the *Optimal Annotator*, which performed well in the analysis of the CREEDS dataset (Fig. 2), enhancing the accuracy and applicability of the framework.

Tang et al. utilized ChatGPT for summarizing medical literature, expressing concerns about the challenge of automatically detecting inconsistent results^17^. To address this, our MRCF implemented a *Quality Controller*, which employs three key criteria to exclude low-quality annotations, thereby achieving high consistency and accuracy. Rather than directly querying ChatGPT, we fed dataset metadata and annotation results into the *Quality Controller*. This method enabled us to maximize the extraction of useful information, resulting in a 95.69% consistency in QC. Furthermore, it automatically identified 15–20% of ChatGPT’s erroneous responses, an aspect not adequately addressed in previous research.

### Advancing transcriptome profiling: overcoming current limits

As of January 13, 2024, the GEO database has incorporated 165,051 transcriptome datasets, extensively mined by numerous teams. Databases like SigCom LINCS, which primarily operate through custom natural language rules, underestimate the complexity of GEO datasets and lack robustness for large-scale clinical application^18^. CREEDS, using manual annotations for pre-training, involves substantial upfront training costs and cannot cover multiple knowledge domains, resulting in lower accuracy^3^. LINCS L1000, while being the largest perturbation study project, only includes actual data for 1058 probes per perturbation, with the remaining 10,000 + gene expressions being inferred, making it difficult to assess the difference between inferred and actual data in practical analysis^19^. Our GEO Auto-Classification Database demonstrates high robustness, powerful knowledge synthesis capabilities, low cost, and high accuracy, all based on real-world data. Additionally, our Drug Repurposing Database facilitates rapid drug screening, offering technical support for precision treatment.It also provides help to quickly build large-scale omics databases like GPSAdb, CMAP, DPNetinfer, etc., for research and application in the medical field^19–21^.

### Current limitations and future prospects

The limitations of this study are mainly threefold. First, although the MRCF excelled in large-scale data mining, it did not encompass all datasets within the GEO database. In the future, we plan to conduct a comprehensive analysis of these datasets to provide rich data in the medical field, support research in areas such as drug repositioning and molecular typing, and apply these SOPs in other data mining domains. Second, despite the gpt4-preview-1106 model's ability to process 128k length tokens, overly long texts might impact quality and lead to misjudgments. We anticipate that the accuracy of long text data annotation will improve as ChatGPT and other LLM technologies evolve. Third, given the diverse and non-standardized nature of the GEO database's data sources, achieving 100% accuracy in data annotation with MRCF remains challenging. Therefore, combining manual and automated analysis becomes crucial to the future work. With complex data sources, the challenges remaininthe need for improved ChatGPT's performance.

Furthermore, we believe this approach and methodology would bring the following breakthroughs in life sciences and big data research: (1) Construction of specific MRCFs for individual domains, (2) Re-mining of difficult-to-organize public data to establish valuable secondary databases, and (3) Research on machine learning algorithms based on large-scale data integration.

## Methods

### Materials

This study utilized 338 manually curated GEO drug perturbation datasets from the CREEDS database as the training set. CREEDS is a knowledge base reliant on crowdsourced microtask projects, focusing on exploring the relationships between drugs, genes, and diseases based on GEO data. The database is characterized by its collection and reanalysis of gene expression profiles, with manually validated unique signatures, but its machine learning annotator has limited effectiveness^3^. Additionally, we conducted a bulk search based on ATC codes and included 782 drugs from the GEO database, totaling 8730 datasets (see Supplementary Table S2). For each drug, we selected 5 datasets for formal analysis (all datasets were included if there were fewer than 5), with detailed methods available in the Supplementary Methods section “Data Collection”. The analysis utilized the latest version of ChatGPT-4 developed by OpenAI (gpt-4-1106-preview API, referred to as ChatGPT), which demonstrates significant advantages in terms of cost, response time, and text processing length (accessed on January 4, 2024, https://platform.openai.com/docs/models/gpt-4-and-gpt-4-turbo). For the complete process’s prompt, see Supplementary Methods S1.

### Pipeline of multi-role ChatGPT framework

We incorporate the Multi-Role Configuration Framework (MRCF), which distinctively addresses the specific challenges of biomedical data annotation. Unlike similar multi-agent LLM frameworks like Microsoft’s AutoGen and Hong et al.’s Data Interpreter, which are primarily optimized for general problem-solving and software engineering tasks, our framework is specialized for effectively handling the intricate dependencies and rigorous standards required in biomedical research^22,23^. This study explores the application of the MRCF solely in the automated mining of the GEO database, yet the framework also holds potential for adaptation to other fields.

The MRCF assigns specific roles within the ChatGPT model framework, optimizing the workflow to enhance efficiency and accuracy as depicted in Fig. 1.

The annotation process begins with the *Original Annotator*, who provides initial annotations based on predefined criteria tailored to biological and chemical entities. These annotations are then evaluated by the *Result Checker*, who compares them against a gold standard to identify discrepancies.

If discrepancies are detected, the *Prompt Optimizer* intervenes by analyzing these discrepancies and suggesting modifications to improve the prompts used by the *Original Annotator*. These refined prompts are reused in the annotation process, and this cycle continues until the annotations achieve the predetermined standard of accuracy.

Upon achieving reliable annotation results, the *Optimal Annotator* applies the refined model across broader datasets. The final annotations are reviewed by the *Quality Controller*, who ensures compliance with three specific criteria: accurate representation of sample identifiers and their characteristics, uniformity in grouping similar samples, and correct assignment of details pertaining to biological perturbations such as type, cell type, and perturbation name. This entire framework ensures the correctness and consistency of the analytical results, providing data support for subsequent differential expression analysis.

### Accuracy evaluation of the multi-role ChatGPT framework

To assess the effectiveness of the MRCF, we conducted a quality evaluation of 317 annotated training sets from the CREEDS database, comparing the consistency between manual and automatic QC of 839 classification results. Manual QC involved comparing the automated annotation results of the *Optimizer Annotator* with the manual annotations from CREEDS. This was divided into three categories: Excellent (where automated annotations were superior to manual), Moderate (equivalent to manual or somewhat ambiguous), and Error (incorrect automated annotations). Automatic QC was conducted using the *Quality Controller*, which was evaluated based on three criteria. Fulfillment of all criteria was marked as Success, otherwise as Fail.

We performed a correlative assessment of both QC results. For instance, datasets marked as “Fail” but manually rated as Excellent indicated that the automated classification was not only correct but also superior to CREEDS’s manual classification. Conversely, datasets marked as “Fail” and manually rated as Error demonstrated that the framework could effectively identify incorrect groupings.

### Consistency and cost–benefit evaluation of the framework

Utilizing the downstream analysis framework of the MRCF, we input 964 drug perturbation datasets into the *Optimal Annotator*. To assess the framework's consistency, we conducted three rounds of QC. Initially, we evaluated the consistency ratio of the first two QCs, and for datasets with inconsistencies, a third round of QC was performed. Finally, datasets that passed the consistency check were used in the automated processing flow and for building the drug database.

In terms of cost, based on the calculation for each dataset containing 2000 words, we compared the pricing of ChatGPT (gpt4-preview-1106 version) with the Google AI Platform Data Labeling Service (https://cloud.google.com/ai-platform/data-labeling/pricing#ai-platform-data-labeling-service-pricing).

### Supplementary Information

Supplementary Information 1.

Supplementary Tables.

## Data Availability

The supplementary data generated and/or analyzed during the current study are available in the Figshare repository, accessible at https://figshare.com/projects/The_metadata_of_MRCF_used_Rdata/204306.

## Code availability

The underlying code for this study is available in the GitHub repository 'Multi_Role_ChatGPT_Framework' and can be accessed via this link: https://github.com/CHR991004/Multi_Role_ChatGPT_Framework.
