# Supplementary material for: Multi role ChatGPT framework for transforming medical data analysis
Source: Sci Rep. 2024 Jun 17;14:13930. doi: 10.1038/s41598-024-64585-5 (PMC11183233; doi:10.1038/s41598-024-64585-5)
Supplement: Supplementary file 1 — Supplementary Information 1. [file 41598_2024_64585_MOESM1_ESM.docx]

**Multi Role ChatGPT Framework for Transforming Medical Data Analysis**

Haoran Chen^1,2†^, Shengxiao Zhang^3,4†^, Lizhong Zhang^5^, Jie Geng^5^, Jinqi Lu^6^, Chuandong Hou^7,8^, Peifeng He^1,9#^ and Xuechun Lu^1,2,7#^

^1^ School of Management, Shanxi Medical University, Taiyuan, 030000, China.

^2^ Department of Nephrology, First Medical Center of Chinese PLA General Hospital, Nephrology Institute of the Chinese People’s Liberation Army, National Key Laboratory of Kidney Diseases, National Clinical Research Center for Kidney Diseases, Beijing Key Laboratory of Kidney Disease Research, Beijing 100853, China

^3^ Department of Rheumatology and Immunology, the Second Hospital of Shanxi Medical University, Taiyuan, China
^4^ Key Laboratory of Coal Environmental Pathogenicity and Prevention at Shanxi Medical University, Ministry of Education, Shanxi Province, Taiyuan, China

^5^ Basic Medicine College, Shanxi Medical University, Taiyuan, 030000, China

^6^ Department of Computer Science, Boston University 665 Commonwealth Avenue, Boston, Massachusetts, 02215, USA

^7^ Department of Hematology, The Second Medical Center of Chinese PLA General Hospital, National Clinical Research Center for Geriatric Disease, Beijing, 100853, China

^8^ Medical School of Chinese PLA, Beijing, 100853, China

^9^ Shanxi Key Laboratory of Big Data for Clinical Decision, Shanxi Medical University, Taiyuan, 030000, China.

^#^ To whom correspondence should be addressed.

^†^ These authors contributed equally: Haoran Chen, Shengxiao Zhang.

**Email:** **Xuechun Lu**: luxuechun@301hospital.com.cn; **Peifeng He:** [hepeifeng2006@126.com](mailto:hepeifeng2006@126.com)

# Methods

## Data Collection

In this study, we collected the names of all drugs recorded in the ATC classification (http://purl.bioontology.org/ontology/ATC/, version 2023AA). Before proceeding with automated data analysis using ChatGPT, it was necessary to organize the data structure and eliminate low-quality and inconsistently formatted data to ensure uniform results. We first established an automated retrieval process for the GEO (Gene Expression Omnibus) ^1^, with the code available at (https://github.com/CHR991004/Multi_Role_ChatGPT_Framework). Specifically, the process included the following steps:

1. The search term format was:

SEARCH-NAME[Title] AND ("Homo sapiens"[porgn] OR "Mus musculus"[porgn] OR "Rattus norvegicus"[porgn]) AND ("gse"[Filter] AND ("Expression profiling by array"[Filter] OR "Expression profiling by high throughput sequencing"[Filter])). Specifically, we filtered out microarray data or RNA-seq data from humans, mice, and rats.

(2) We used the rentrez package to retrieve corresponding dataset information such as Entrez ID, GSE ID, GPL ID, title, and abstract^2^.

(3) To increase efficiency, we employed R language code to automatically convert GSE IDs into download links (the downloaded files contain metadata for each sample in the GSE dataset).

(4) We used the GEOquery (v2.68.0) package to read the downloaded data into R software in a standardized format, serving as input data for the subsequent ChatGPT process.

(5) If the species were mice or rats, we used the biomaRt package to uniformly convert their gene IDs into HGNC gene symbols.

(6) For datasets of the "Expression profiling by array" type, we used the standardized series matrix from the GEO database. For datasets of the "Expression profiling by high throughput sequencing" type, we used the count expression matrices processed by the GREIN database or the GEO website, which were then utilized for subsequent differential expression analysis.

## Optimization of Original Prompts

In this study, we employed a negative feedback iterative algorithm, using GSE numbers as input and an *Original Annotator*, based on our empirical, to annotate GSE dataset samples. Subsequently, the annotations generated by ChatGPT were compared with the expert's gold standard annotations (obtained from CREEDS) to optimize any deficiencies. This process was repeated until the *Optimal Annotator* was developed, which was then used for data classification (see **Conversation Example**). To prevent ChatGPT from producing random outputs and entering an endless iteration loop, we introduced a termination command: “NO_CHANGE_REQUIRED.” When ChatGPT determines that no further optimization is needed for the *Original Annotator*, it automatically stops iterating and retains the optimal prompt.

## Construction of an Automated Classification System Based on the Optimal Annotator

To efficiently utilize the omics data in the GEO database for clinical analysis, we standardized the recording of six key pieces of information based on the Metadata of the obtained GSE dataset and the Optimal prompt: GSE id, cell type, control group id, perturbation group id, perturbation type, perturbation name. This facilitates the rapid construction of an omics database. Additionally, considering biological significance and result randomness, we implemented preliminary quality control on the annotated results, including: (1) a maximum of six re-analyses; (2) re-analysis if the result table is not in six columns; (3) re-analysis if the result is empty; (4) deletion of rows in the control group id and perturbation id that do not start with GSM; (5) deletion of rows where the GSM id count is less than three. Results that do not pass quality control are retained for manual review to decide whether to re-analyze or discard them.

Specifically, we limited ChatGPT's return standards to enable the framework to accurately recognize and use regular expressions for standardization: “Create a table without column names. Use a tab character (\t) to separate between conditions and reasons, and a newline character (\n) to separate rows. The first column must list with the format in parentheses: (condition 1(+/-), condition 2(+/-), condition 3(+/-)), where a '+' indicates the condition is met and a '-' indicates it is not. The second column should contain the reasons for the three conditions (If there is a '-' in the condition, it indicates a reason; otherwise, use single 'NA' as a placeholder, 3 conditions separate with ','). Do not include any additional explanations or information in the response.”

Finally, we also used regular expressions to standardize ChatGPT's output results to ensure quality. Data that did not meet the standards were re-analyzed, with the final results used for data analysis.

## Automated Transcriptome Data Differential Expression Analysis

Annotations only recorded the GSM IDs of the perturbation and control groups, but practical application also requires identification of differentially expressed genes (DEGs) post-perturbation. Therefore, we designed an automatic analysis process, wherein the program obtains annotations and gene expression matrices, and uses two different algorithms based on sequencing principles to obtain DEGs of perturbations. All analyses follow a unified standard, with a threshold of *P* < 0.05, |log2FC| > |log2FC|mean + 2 × |log2FC|SD. We use the GEOquery (version 2.68.0) package to obtain expression matrices for microarray data and rely on GREIN (<http://www.ilincs.org/apps/grein>) and GEO2R (<https://www.ncbi.nlm.nih.gov/geo/geo2r>) for the quantitative count matrices of RNA-seq. For microarray data, we use the biomaRt (version 2.56.1) package to convert gene symbols of rats and mice into human gene symbols. The impute (version 1.74.1) package is employed to impute missing expression data, and the limma (version 3.56.2) package for data normalization and differential expression analysis^3^. For high throughput RNA-seq data, we also use biomaRt for converting non-human gene IDs into human gene symbols, followed by differential expression analysis using the DESeq2 (version 1.40.2) package^4^. Benefiting from the standardized outputs provided by the Multi-Role ChatGPT Framework, this method facilitates a completely automated process for analyzing differential gene expression. For in-depth information and access to the specific code, please visit <https://github.com/CHR991004/Multi_Role_ChatGPT_Framework>.

## Development and Application of Databases

We plan to establish two databases for research experts to use without the need for programming skills. First, the foundational database, DataSciChat, extracts metadata from the GEO database using the R language’s GEOquery package. It then annotates, quality controls, and re-analyzes datasets within the Multi-Role ChatGPT Framework. All operations are based on the pre-set *Optimal Annotator* to ensure accuracy. Users can input a GSE ID list to process and retrieve all results in bulk, storing the quality control-approved results using the RSQLite (version 2.3.1) package, thereby reducing costs and resource usage.

The second database is the application-oriented Drug Repurposing Database. Utilizing perturbation datasets that have undergone preliminary quality control and differential expression analysis, we created a database with drug perturbation signatures search capability using the signatureSearch package (version 1.16.0)^5.^ Users can input custom lists of differentially expressed genes, including gene names and log fold change values, to filter drugs with similar or opposing expression patterns and identify potential targets. To validate this database, we conducted analyses and verifications using datasets from Acute Myeloid Leukemia (GSE116616) and the drug Imatinib (GSE33075).

# Conversation Example

To comprehensively demonstrate the analysis process, we use GSE7036 as a case study. Considering the substantial amount of text involved, this section is presented in textual form instead of screenshots and involves the Original Annotator, Prompt Optimizer, Optimal Annotator, and Quality Controller. Detailed code and information about the prompts can be found at <https://github.com/CHR991004/Multi_Role_ChatGPT_Framework>.

## Original Annotator

### User:

I am conducting a bioinformatics analysis and need assistance with automated sample grouping for differential expression analysis. Here are the details:\ngse_id: GSE7036\nabstract: To identify genes dysregulated in bipolar disorder (BD1) we carried out global gene expression profiling using whole-genome microarrays. To minimize genetic variation in gene expression levels between cases and controls we compared expression profiles in lymphoblastoid cell lines from monozygotic twin pairs discordant for the disease. We identified 82 genes that were differentially expressed by ≥ 1.3-fold in 3 BD1 cases compared to their co-twins, and which were statistically (p ≤ 0.05) differentially expressed between the groups of BD1 cases and controls. Using qRT-PCR we confirmed the differential expression of some of these genes, including: KCNK1, MAL, PFN2, TCF7, PGK1, and PI4KCB, in at least 2 of the twin pairs. In contrast to the findings of a previous study by Kakiuchi and colleagues with similar discordant BD1 twin design1 our data do not support the dysregulation of XBP1 and HSPA5. From pathway and gene ontology analysis we identified up-regulation of the WNT signalling pathway and the biological process of apoptosis. The differentially regulated genes and pathways identified in this study may provide insights into the biology of BD1. \nKeywords: Bipolar Disorder, Monozygotic Twins, Discordant, Trasnsformed Lymphocytes\nExperimental design: Three pairs of Monozygotic Twins which were discordant for Bipolar Disorder with psychotic features were examined. (n=6) \nSample info: Trasnsformed Lymphocytes Bipolar Disorder Affected Twin Pair 1 (DCBD1.1)(TP5)\tGSM162897\tSample ID:TP5\tTwin Pair ID:DCBD1.1\tClinical Status:BD1 with psychotic features\tAge of Onset:19\tAge at interview & blood sampling:55\tCurrent Age (2007):58\tSex: Female\tMedication at interview (duration) :lithium & citalopram (22.5 years)\n\nSample info: Trasnsformed Lymphocytes Unaffected Twin Pair 1(DCBD1.2)(TP6)\tGSM162898\tSample ID:TP6\tTwin Pair ID:DCBD1.2\tClinical Status:Control \tAge of Onset:-\tAge at interview & blood sampling:55 \tCurrent Age (2007):58 \tSex: Female \tMedication at interview (duration) :None\n\nSample info: Trasnsformed Lymphocytes Bipolar Disorder Affected Twin Pair 2 (DCBD2.1)(TP7)\tGSM162899\tSample ID:DCBD2.1 \tTwin Pair ID:TP7 \tClinical Status:BD1 with psychotic features \tAge of Onset:19 \tAge at interview & blood sampling:20 \tCurrent Age (2007):24\tSex: Male \tMedication at interview (duration) :lithium & olanzapine (64 weeks)\n\nSample info: Trasnsformed Lymphocytes Unaffected Twin Pair 2 (DCBD2.2)(TP8)\tGSM162900\tSample ID:DCBD2.1 \tTwin Pair ID:TP8 \tClinical Status:Control\tAge of Onset:-\tAge at interview & blood sampling:20 \tCurrent Age (2007):24\tSex: Male \tMedication at interview (duration) :none\n\nSample info: Trasnsformed Lymphocytes Bipolar Disorder Affected Twin Pair 3 (DCBD3.1)(TP9)\tGSM162901\tSample ID:TP9\tTwin Pair ID:DCBD3.1\tClinical Status:BD1 with psychotic features \tAge of Onset:18 \tAge at interview & blood sampling:35 \tCurrent Age (2007):38 \tSex: Female \tMedication at interview (duration) :sodium valproate (5 years)\n\nSample info: Trasnsformed Lymphocytes Unaffected Twin Pair 3 (DCBD3.2)(TP10)\tGSM162903\tSample ID:TP10 \tTwin Pair ID:DCBD3.2 \tClinical Status:Control\tAge of Onset:-\tAge at interview & blood sampling:35 \tCurrent Age (2007):38 \tSex: Female \tMedication at interview (duration) :None \n All requirements you need to follow:Generate a tab-separated table based on the provided sample information. The table should strictly contain the following columns only/: gse_id, cell_type, ctrl_ids, pert_ids, type, and pert_name. Each row must represent a unique perturbation. Use '\t' for column separation and '|' to separate multiple GSM ids within the same cell. Please ensure the response contains only the table, with no introductory, explanatory, or additional text. Inclusion Criteria:\tIf all samples do not meet the criteria for comparison or there are no biological duplicates, only the GSE_ID will be returned.\tExclude comparisons between control groups.\tColumns and Content:\tThe table should include columns for GSE_id (or geo_id), cell_type, ctrl_ids (control GSM id), pert_ids (perturbation GSM id), type (restricted to 'disease', 'drug', 'gene'), and pert_name (a detailed name for the perturbation).\tThe 'pert_name' should represent a single perturbation factor, not a combination.\tSpecificity in Naming and Grouping:\tFor cell_type, use specific terms instead of general terms like .\tPerturbations involving a combination of factors should be separated into individual rows rather than combined.\tAdditional Information:\tRecord specific tissue or cell line from the original sample, along with the time for each perturbation.\tUse 'NA' where information is unavailable.\tNo extraneous text should be included outside of the table format.

### ChatGPT Answer:

GSE7036\tTransformed Lymphocytes\tGSM162898|GSM162897\tdisease\tBipolar Disorder with psychotic features\nGSE7036\tTransformed Lymphocytes\tGSM162900\t GSM162899\tdisease\tBipolar Disorder with psychotic features\n GSE7036\tTransformed Lymphocytes\tGSM162903\t GSM162901\tdisease\tBipolar Disorder with psychotic features

## Prompt Optimizer

Due to the multi-turn optimizer requirement of ChatGPT Prompt, and the randomness in ChatGPT responses, this section only displays the initial input and the final output.

### User:

(Required: Directly provide me with the prompt content without any description or introduction)As a quality assurance engineer, I am reviewing the output of a ChatGPT-based classification system for bioinformatics data. Below are the details:\n\nFixed Original Prompt:\nAll requirements you need to follow:Generate a tab-separated table based on the provided sample information. The table should strictly contain the following columns only/: gse_id, cell_type, ctrl_ids, pert_ids, type, and pert_name. Each row must represent a unique perturbation. Use '\t' for column separation and '|' to separate multiple GSM ids within the same cell. Please ensure the response contains only the table, with no introductory, explanatory, or additional text.Modifiable Original Prompt:\nInclusion Criteria:\tIf all samples do not meet the criteria for comparison or there are no biological duplicates, only the GSE_ID will be returned.\tExclude comparisons between control groups.\tColumns and Content:\tThe table should include columns for GSE_id (or geo_id), cell_type, ctrl_ids (control GSM id), pert_ids (perturbation GSM id), type (restricted to 'disease', 'drug', 'gene'), and pert_name (a detailed name for the perturbation).\tThe 'pert_name' should represent a single perturbation factor, not a combination.\tSpecificity in Naming and Grouping:\tFor cell_type, use specific terms instead of general terms like .\tPerturbations involving a combination of factors should be separated into individual rows rather than combined.\tAdditional Information:\tRecord specific tissue or cell line from the original sample, along with the time for each perturbation.\tUse 'NA' where information is unavailable.\tNo extraneous text should be included outside of the table format.\n\ngse_id: GSE7036\nabstract: To identify genes dysregulated in bipolar disorder (BD1) we carried out global gene expression profiling using whole-genome microarrays. To minimize genetic variation in gene expression levels between cases and controls we compared expression profiles in lymphoblastoid cell lines from monozygotic twin pairs discordant for the disease. We identified 82 genes that were differentially expressed by ≥ 1.3-fold in 3 BD1 cases compared to their co-twins, and which were statistically (p ≤ 0.05) differentially expressed between the groups of BD1 cases and controls. Using qRT-PCR we confirmed the differential expression of some of these genes, including: KCNK1, MAL, PFN2, TCF7, PGK1, and PI4KCB, in at least 2 of the twin pairs. In contrast to the findings of a previous study by Kakiuchi and colleagues with similar discordant BD1 twin design1 our data do not support the dysregulation of XBP1 and HSPA5. From pathway and gene ontology analysis we identified up-regulation of the WNT signalling pathway and the biological process of apoptosis. The differentially regulated genes and pathways identified in this study may provide insights into the biology of BD1. \nKeywords: Bipolar Disorder, Monozygotic Twins, Discordant, Trasnsformed Lymphocytes\nExperimental design: Three pairs of Monozygotic Twins which were discordant for Bipolar Disorder with psychotic features were examined. (n=6) Original Output (before any prompt modification): \nGSE7036\tTransformed Lymphocytes\tGSM162898\tGSM162897\tdisease\tBipolar Disorder with psychotic features\nGSE7036\tTransformed Lymphocytes\tGSM162900\tGSM162899\tdisease\tBipolar Disorder with psychotic features\nGSE7036\tTransformed Lymphocytes\tGSM162903\tGSM162901\tdisease\tBipolar Disorder with psychotic features\nTarget \ngeo_id\tcell_type\tctrl_ids\tpert_ids\ttype\tpert_name\nGSE7036\tLymphocyte\tGSM162898|GSM162900|GSM162903\tGSM162897|GSM162899|GSM162901\tdisease\tbipolar disorder\nGSE7036\tlymphoblastoid cells\tGSM162898|GSM162900|GSM162903\tGSM162897|GSM162899|GSM162901\tdrug\tCitalopram\n\n1. Assess the relationship between original_output and target_output to determine if the 'prompt' needs adjustments (You do not need to tell me whether adjustments is needed just return prompt or \"NO_CHANGE_REQUIRED\".).\t(2. If the original output is the same, similar, or better than the target output, no adjustments are needed. In this case, respond only with \"NO_CHANGE_REQUIRED\".)\t(3. If adjustments are necessary(Please ensure the response contains only the prompt, with no introductory, explanatory, or additional text.):\t3.1.Include the original prompt (original_prompt).\t3.2. Cannot write any examples (For example, the name of a specific drug, a disease, or a cell line) in prompt, but put the summarized rules that need to be optimized into prompt!\t3.3.Put the newly added requirements after original prompt.\t3.4.Please ensure that the supplemental prompt is as concise as possible.)

### ChatGPT Answer:

All requirements you need to follow: Generate a tab-separated table based on the provided sample information. The table must strictly contain the following columns only/:gse_id, cell_type, ctrl_ids, pert_ids, type(According to the description, categorize into gene/disease/drug/other.If it's definitely not possible to categorize, only then classify as 'other'.), and pert_name. Each row must represent a unique perturbation. Use '\t' for column separation and'' to separate multiple GSM ids within the same cell. Please ensure the response contains only the table, with no introductory, explanatory, or additional text. Additional Requirements:1.If the patient is on medication, the type is the drug 2.single ctrl_ids and pert_ids must be IDs starting with GSM 3.If the sample description is vague, you can refer to abstract 4.If the type is a drug, the pert_name should specify the drug name, dosage, and duration of action (if applicable, Format is drug(dose, time)) 5.The result must return the full name of the drug (if applicable)rather than an abbreviation.

## Optimal Annotator

### User:

I am conducting a bioinformatics analysis and need assistance with automated sample grouping for differential expression analysis. Here are the details:\ngse_id: GSE7036\nabstract: To identify genes dysregulated in bipolar disorder (BD1) we carried out global gene expression profiling using whole-genome microarrays. To minimize genetic variation in gene expression levels between cases and controls we compared expression profiles in lymphoblastoid cell lines from monozygotic twin pairs discordant for the disease. We identified 82 genes that were differentially expressed by ≥ 1.3-fold in 3 BD1 cases compared to their co-twins, and which were statistically (p ≤ 0.05) differentially expressed between the groups of BD1 cases and controls. Using qRT-PCR we confirmed the differential expression of some of these genes, including: KCNK1, MAL, PFN2, TCF7, PGK1, and PI4KCB, in at least 2 of the twin pairs. In contrast to the findings of a previous study by Kakiuchi and colleagues with similar discordant BD1 twin design1 our data do not support the dysregulation of P1 and HSPA5. From pathway and gene ontology analysis we identified up-regulation of the WNT signalling pathway and the biological process of apoptosis. The differentially regulated genes and pathways identified in this study may provide insights into the biology of BD1. \nKeywords: Bipolar Disorder, Monozygotic Twins, Discordant, Trasnsformed Lymphocytes\nExperimental design: Three pairs of Monozygotic Twins which were discordant for Bipolar Disorder with psychotic features were examined. (n=6) \nSample info: title\tgeo_accession\tsource_name_ch1\tcharacteristics_ch1\tcharacteristics_ch1.1\tcharacteristics_ch1.2\tcharacteristics_ch1.3\tcharacteristics_ch1.4\tcharacteristics_ch1.5\tcharacteristics_ch1.6\tcharacteristics_ch1.7\tdescription\n\nSample info: Trasnsformed Lymphocytes Bipolar Disorder Affected Twin Pair 1 (DCBD1.1)(TP5)\tGSM162897\tTrasnsformed Lymphocytes Bipolar Disorder Affected (Affected pateint of MZ twin pair 1)\tSample ID:TP5\tTwin Pair ID:DCBD1.1\tClinical Status:BD1 with psychotic features\tAge of Onset:19\tAge at interview & blood sampling:55\tCurrent Age (2007):58\tSex: Female\tMedication at interview (duration) :lithium & citalopram (22.5 years)\t-\n\nSample info: Trasnsformed Lymphocytes Unaffected Twin Pair 1(DCBD1.2)(TP6)\tGSM162898\tTrasnsformed Lymphocytes Unaffected (Unaffected pateint of MZ twin pair 1)\tSample ID:TP6\tTwin Pair ID:DCBD1.2\tClinical Status:Control \tAge of Onset:-\tAge at interview & blood sampling:55 \tCurrent Age (2007):58 \tSex: Female \tMedication at interview (duration) :None\t-\n\nSample info: Trasnsformed Lymphocytes Bipolar Disorder Affected Twin Pair 2 (DCBD2.1)(TP7)\tGSM162899\tTrasnsformed Lymphocytes Bipolar Disorder Affected (Affected pateint of MZ twin pair 2)\tSample ID:DCBD2.1 \tTwin Pair ID:TP7 \tClinical Status:BD1 with psychotic features \tAge of Onset:19 \tAge at interview & blood sampling:20 \tCurrent Age (2007):24\tSex: Male \tMedication at interview (duration) :lithium & olanzapine (64 weeks)\t-\n\nSample info: Trasnsformed Lymphocytes Unaffected Twin Pair 2 (DCBD2.2)(TP8)\tGSM162900\tTrasnsformed Lymphocytes Unaffected (Unaffected pateint of MZ twin pair 2)\tSample ID:DCBD2.1 \tTwin Pair ID:TP8 \tClinical Status:Control\tAge of Onset:-\tAge at interview & blood sampling:20 \tCurrent Age (2007):24\tSex: Male \tMedication at interview (duration) :none\t-\n\nSample info: Trasnsformed Lymphocytes Bipolar Disorder Affected Twin Pair 3 (DCBD3.1)(TP9)\tGSM162901\tTrasnsformed Lymphocytes Bipolar Disorder Affected (Affected pateint of MZ twin pair 3)\tSample ID:TP9\tTwin Pair ID:DCBD3.1\tClinical Status:BD1 with psychotic features \tAge of Onset:18 \tAge at interview & blood sampling:35 \tCurrent Age (2007):38 \tSex: Female \tMedication at interview (duration) :sodium valproate (5 years)\t-\n\nSample info: Trasnsformed Lymphocytes Unaffected Twin Pair 3 (DCBD3.2)(TP10)\tGSM162903\tTrasnsformed Lymphocytes Unaffected (Unaffected pateint of MZ twin pair 3)\tSample ID:TP10 \tTwin Pair ID:DCBD3.2 \tClinical Status:Control\tAge of Onset:-\tAge at interview & blood sampling:35 \tCurrent Age (2007):38 \tSex: Female \tMedication at interview (duration) :None\t- \n All requirements you need to follow:Generate a tab-separated table based on the provided sample information. The table must strictly contain the following columns only/: gse_id, cell_type, ctrl_ids, pert_ids, type(According to the description, categorize into gene/disease/drug/other. If it's definitely not possible to categorize, only then classify as 'other'.), and pert_name. Each row must represent a unique perturbation. Use '\t' for column separation and '|' to separate multiple GSM ids within the same cell. Please ensure the response contains only the table, with no introductory, explanatory, or additional text. Additional Requirements:1.Type refers to the drug only if the patient is medicated and the drug is a controlled variable.\n2.Single ctrl_ids and pert_ids must be IDs starting with GSM and not text descriptions.\n3.If the sample description is vague, you can refer to abstract\n4.If the type is a drug, the pert_name should specify the drug name, dosage, and duration of action (if applicable,Format is drug(dose, time))\n5.The result must return the full name of the drug (if applicable) rather than an abbreviation.

### ChatGPT Answer:

GSE7036\tTrasnsformed Lymphocytes\tGSM162898\tGSM162897\tdisease\tBipolar Disorder with psychotic features\nGSE7036\tTrasnsformed Lymphocytes\tGSM162900\tGSM162899\tdisease\tBipolar Disorder with psychotic features\n GSE7036\tTrasnsformed Lymphocytes\tGSM162903\tGSM162901\tdisease\tBipolar Disorder with psychotic features\n GSE7036\tTrasnsformed Lymphocytes\tGSM162898\tGSM162897\tdrug\tlithium & citalopram (22.5 years) \n GSE7036\tTrasnsformed Lymphocytes\tGSM162900\tGSM162899\tdrug\tlithium & olanzapine (64 weeks) \n GSE7036\tTrasnsformed Lymphocytes\tGSM162903\tGSM162901\tdrug\tsodium valproate (5 years)

## Quality Controller

### User:

You are a statistician, biologist and pharmacy expert who need assistance to check if the grouping is correct.I am seeking a detailed and careful analysis of my data for quality control. Focusing on three conditions : Condition 1. Please verify and double-check the GSM details for both control_ids and pert_ids, ensuring alignment with their respective GSM characteristics because you are very easy to make mistakes here.In each row, Both the control group (ctrl_ids) and the perturbation roup (pert_ids) adhere to principle of controlling variables, where all variables(you must Ignore any sex or age mismatch between pert_ids and ctrl_ids.) except one are kept constant. Moreover,the unequal sample sizes between the pert and control groups are permissible.Condition 2. All same types of samples are grouped into one grid cell. Pay attention to the classification in the \"type\" column. Condition 3. Verify whether pert(drug/disease/gene) cell_type and pert_name (different conditions between pert_ids and ctrl_ids) is correct. Your Answer format: (Create a table without column names. Use a tab character ('\\t') to separate between conditions and reasons, and a newline character ('\\n') to separate rows. The first column must list with the format in parentheses: (condition 1(+/-), condition 2(+/-), condition 3(+/-)), where a '+' indicates the condition is met and a '-' indicates it is not. The second column should contain the reasons for the three conditions (If there is a '-' in the condition, it indicates a reason; otherwise, use single 'NA' as a placeholder, 3 conditions separate with ','). Do not include any additional explanations or information in the response..\nHere are my grouping details(Note:You should convert to the table format to understand the following information,'\\t' is a tab and '\\n' is a line break,The entries in the Control IDs (ctrl_ids) and Perturbation IDs (pert_ids) are separated by vertical bars (|).VERY IMPORTANT: I only Input one line, so you only need to return the one row): (V1\tV2\tV3\tV4\tV5\tV6\nGSE7036\tTransformed Lymphocytes\tGSM162898\tGSM162897\tdisease\tBipolar Disorder)The information you can refer to is as following:\n---\nExperimental Abstract(abstract is only used for background knowledge introduction and not suitable for grouping or condition evaluation): To identify genes dysregulated in bipolar disorder (BD1) we carried out global gene expression profiling using whole-genome microarrays. To minimize genetic variation in gene expression levels between cases and controls we compared expression profiles in lymphoblastoid cell lines from monozygotic twin pairs discordant for the disease. We identified 82 genes that were differentially expressed by ≥ 1.3-fold in 3 BD1 cases compared to their co-twins, and which were statistically (p ≤ 0.05) differentially expressed between the groups of BD1 cases and controls. Using qRT-PCR we confirmed the differential expression of some of these genes, including: KCNK1, MAL, PFN2, TCF7, PGK1, and PI4KCB, in at least 2 of the twin pairs. In contrast to the findings of a previous study by Kakiuchi and colleagues with similar discordant BD1 twin design1 our data do not support the dysregulation of XBP1 and HSPA5. From pathway and gene ontology analysis we identified up-regulation of the WNT signalling pathway and the biological process of apoptosis. The differentially regulated genes and pathways identified in this study may provide insights into the biology of BD1. \nKeywords: Bipolar Disorder, Monozygotic Twins, Discordant, Trasnsformed Lymphocytes\n---\nCharacteristics of each GSM (Table format,Separator with '\\t' and newline with '\\n',):\nTrasnsformed Lymphocytes Bipolar Disorder Affected Twin Pair 1 (DCBD1.1)(TP5)\tGSM162897\tSample ID:TP5\tTwin Pair ID:DCBD1.1\tClinical Status:BD1 with psychotic features\tAge of Onset:19\tAge at interview & blood sampling:55\tCurrent Age (2007):58\tSex: Female\tMedication at interview (duration) :lithium & citalopram (22.5 years)\nTrasnsformed Lymphocytes Unaffected Twin Pair 1(DCBD1.2)(TP6)\tGSM162898\tSample ID:TP6\tTwin Pair ID:DCBD1.2\tClinical Status:Control \tAge of Onset:-\tAge at interview & blood sampling:55 \tCurrent Age (2007):58 \tSex: Female \tMedication at interview (duration) :None

### ChatGPT Answer:

(condition 1(+), condition 2(+), condition 3(+)) NA, NA, NA

# Reference

1 Clough, E. *et al.* NCBI GEO: archive for gene expression and epigenomics data sets: 23-year update. *Nucleic acids research* **52**, D138-d144, doi:10.1093/nar/gkad965 (2024).

2 Winter, D. J. rentrez: An R package for the NCBI eUtils API. Report No. 2167-9843, (PeerJ Preprints, 2017).

3 Ritchie, M. E. *et al.* limma powers differential expression analyses for RNA-sequencing and microarray studies. *Nucleic acids research* **43**, e47, doi:10.1093/nar/gkv007 (2015).

4 Love, M. I., Huber, W. & Anders, S. Moderated estimation of fold change and dispersion for RNA-seq data with DESeq2. *Genome biology* **15**, 550, doi:10.1186/s13059-014-0550-8 (2014).

5 Duan, Y. *et al.* signatureSearch: environment for gene expression signature searching and functional interpretation. *Nucleic acids research* **48**, e124, doi:10.1093/nar/gkaa878 (2020).
